# Rational Design of Covalent Organic Frameworks-Based Single Atom Catalysts for Oxygen Evolution Reaction and Oxygen Reduction Reaction

**DOI:** 10.3390/molecules30071505

**Published:** 2025-03-28

**Authors:** Wenli Xie, Bin Cui, Desheng Liu, Haicai Huang, Chuanlu Yang

**Affiliations:** 1 School of Materials Science and Engineering, Guangdong Ocean University, Yangjiang 529500, China; wlxie@gdou.edu.cn; 2 State Key Laboratory of Crystal Materials, School of Physics, Shandong University, Jinan 250100, China; cuibin@sdu.edu.cn (B.C.); liuds@sdu.edu.cn (D.L.); 3 Chongqing Institute of Green and Intelligent Technology, Chinese Academy of Sciences, Chongqing 400714, China; 4 Chongqing School, University of Chinese Academy of Sciences, Chongqing 400714, China; 5 School of Physics and Optocelectronic Engineering, Ludong University, Yantai 264025, China

**Keywords:** single atom catalysts, covalent organic frameworks, oxygen evolution reaction, oxygen reduction reaction, first principles calculation

## Abstract

The rational design of high-performance catalysts for the oxygen evolution reaction (OER) and oxygen reduction reaction (ORR) is essential for the development of clean and renewable energy technologies, particularly in fuel cells and metal-air batteries. Two-dimensional (2D) covalent organic frameworks (COFs) possess numerous hollow sites, which contribute to the stable anchoring of transition metal (TM) atoms and become promising supports for single atom catalysts (SACs). Herein, the OER and ORR catalytic performance of a series of SACs based on TQBQ-COFs were systematically investigated through density functional theory (DFT) calculations, with particular emphasis on the role of the coordination environment in modulating catalytic activity. The results reveal that Rh/TQBQ exhibits the most effective OER catalytic performance, with an overpotential of 0.34 V, while Au/TQBQ demonstrates superior ORR catalytic performance with an overpotential of 0.50 V. A critical mechanistic insight lies in the distinct role of boundary oxygen atoms in TQBQ, which perturb the adsorption energetics of reaction intermediates, thereby circumventing conventional scaling relationships governing OER and ORR pathways. Furthermore, we established the adsorption energy of TM atoms (Ead) as a robust descriptor for predicting catalytic activity, enabling a streamlined screening strategy for SAC design. This study emphasizes the significance of the coordination environment in determining the performance of catalysts and offers a new perspective on the design of novel and effective OER/ORR COFs-based SACs.

## 1. Introduction

The escalating global energy demand, coupled with pressing environmental challenges, underscores the urgent need to transition toward clean and renewable energy systems [1,2,3,4]. Among emerging clean energy technologies, metal-air batteries and fuel cells have garnered extensive attention in recent years due to their high energy density and zero-emission potential [5,6]. The oxygen evolution reaction (OER) and oxygen reduction reaction (ORR) are crucial chemical reactions within these energy conversion devices, but are limited by sluggish kinetic processes [7,8]. Accordingly, the development of catalysts with high activity to improve the reaction rate is essential to overcome this dilemma. Traditional strategies to optimize catalytic performance have focused on nanostructuring to maximize active site exposure, yet these approaches often face limitations in stability and atomic utilization efficiency.

Single-atom catalysts (SACs), which are composed of individual atoms dispersed on diverse substrates, provide a distinctive strategy to maximize atomic efficiency and ensure the uniform distribution of active sites [9]. Attributable to their homogeneity, SACs can achieve excellent catalytic selectivity, offering flexibility to target specific products and activities through accurate single-atom distribution on solid supports. Currently, SACs have been widely used in various chemical reactions, encompassing hydrogen evolution, oxygen evolution, oxygen reduction, nitrogen reduction, and carbon dioxide reduction reactions and so on [10,11,12,13,14,15]. SACs are highly sensitive to the local coordination environment and the chemical properties of coordination atoms, leading to potentially significant variations in the catalytic properties of different metal atoms supported on the same substrate, which highlights the versatility and selectivity of SACs. Previous studies have shown that some two-dimensional materials, such as graphene, layered double hydroxides (LDHs), carbon nitride, transition metal dihalides (TMDs), and MXenes, are the main supports for SACs due to their structural stability and ease of preparation [16,17,18,19,20]. The atomic loading and stability of catalysts have always been the main challenges in the synthesis of SACs. Consequently, the quest for optimal SAC supports remains a pivotal challenge.

Covalent organic frameworks (COFs), with their periodic porous architectures and abundant heteroatom-rich coordination sites, offer a compelling alternative. This special structure endows COFs have strong binding ability with metal atoms, resulting in special heterogeneous catalysts. Experimentally, many COFs-supported SACs have been successfully synthesized, and their high catalytic activity has also been established. For instance, based on DFT calculations, Zeng et al. screened two systems, Co-COF-C4N and Ni-COF-C4N, and considered them to be highly active and low-cost OER SACs for target synthesis [21]. Their subsequent experiments confirmed the excellent OER activity of Co-COF-C4N with an overpotential of 280 mV at 10 mA cm^−2^, which is superior to most of the previously reported OER electrocatalysts. Separately, Zhang et al. prepared single-atom platinum (Pt) with high loading content anchored on the pore walls of two-dimensional β-ketoenamine-linked COF (TpPa-1-COF). The optimized Pt1@TpPa-1 catalyst exhibited a high photocatalytic H_2_ evolution rate of 719 μmol g^−1^ h^−1^ with a high actual Pt loading content of 0.72 wt% [22]. In order to effectively screen 2D COFs-based SACs, theoretical research workers have also conducted systematic studies. Li et al. focused on studying the binding sites and stability of Pd single atoms (SAs) dispersed on triazine COF (Pd1/trzn-COF), along with the reaction mechanism of CO oxidation [23]. A number of transition metal (TM) atoms embedded on a 2D COF Pc-TFPN (TMPc-TFPN) as SACs for ammonia synthesis under ambient conditions were investigated by Zhao et al. [24]. These theoretical and experimental research works have jointly confirmed the immense potential of COF materials as efficient SACs supports. Despite these advances, mechanistic insights into how boundary coordination environments—particularly unsaturated heteroatoms—modulate catalytic behavior remain underexplored. In fact, the adjacent unsaturated coordination atoms have an important influence on catalytic performance. Chen et al. successfully synthesized COFs containing triquinoxalinylene and benzoquinone units, called TQBQ-COF [25]. TQBQ-COF not only possesses large regular pores but also has a large number of unsaturated coordinated N and O atoms at the edge, making it a promising candidate material for embedding metal atoms. Consequently, we aimed to explore the feasibility of using TQBQ-COF as a SAC support to realize efficient OER/ORR catalysis. We further investigated the important role of boundary unsaturated atoms in the catalytic process.

In this study, we investigated the catalytic performance of TM atoms supported on TQBQ-COF (TM/TQBQ) for OER/ORR based on the DFT method. The unsaturated coordination sites at the TQBQ-COF periphery not only stabilize TM atoms but also actively participate in intermediate adsorption, thereby disrupting conventional scaling relationships that limit OER/ORR efficiency. Through charge analysis and the calculation of the density of states (DOS), we explored the activity origin of the catalyst, emphasizing the key role of coordination atoms in determining catalytic performance. In addition, we established the scaling relationship between the adsorption energy of metal atoms (*E*_ad_), the transfer charge (∆*q*), and the adsorption energy of intermediates. The results reveal that these parameters can well describe the catalytic activity of the reaction. Our work provides a new perspective for screening effective OER/ORR catalysts and lays a solid theoretical foundation for the design of COFs-based SACs.

## 2. Computational Details

The spin polarization DFT calculations were performed as implemented in the Vienna Ab initio Simulation Package (VASP 4.6) [26] using the projector augmented wave (PAW) potential [27]. To describe the electronic exchange-correlation interactions, the generalized gradient approximation (GGA) method with Perdew–Burke–Ernzerhof (PBE) functional was used [28]. C 2s^2^2p^2^, N 2s^2^2p^3^, and O N 2s^2^2p^4^ were treated as the valence states. The electron energy level was occupied by Gaussian smearing with a width of σ = 0.05 eV. A kinetic energy cutoff of 450 eV for electron wave expansion was used. The finite displacement method was used for structure optimization, and the converge criteria for energy and residual force were set to 10^−5^ eV and 0.02 eV/Å, respectively. The k-point meshes were selected to be 5 × 5 × 1 and 7 × 7 × 1 for the optimization of the structures and the calculation of electronic structures, respectively. A vacuum layer of 16 Å along the z-direction was adopted to avoid layer interactions. The van der Waals (VDW) interaction was described by the semiempirical correction scheme of the Grimme (DFT-D3) method throughout the calculation [29]. Materials Studio (MS 6.0) software was used for structural modeling and charge density difference analysis was performed within Visualization for Electronic and Structural Analysis (VESTA) [30], and vaspkit script was used for electronic structural analysis and processing [31]. More computational details can be found in the Supplementary Materials.

## 3. Results and Discussion

### 3.1. Structural Stability and Electronic Properties of TM/TQBQ

**Figure 1:**
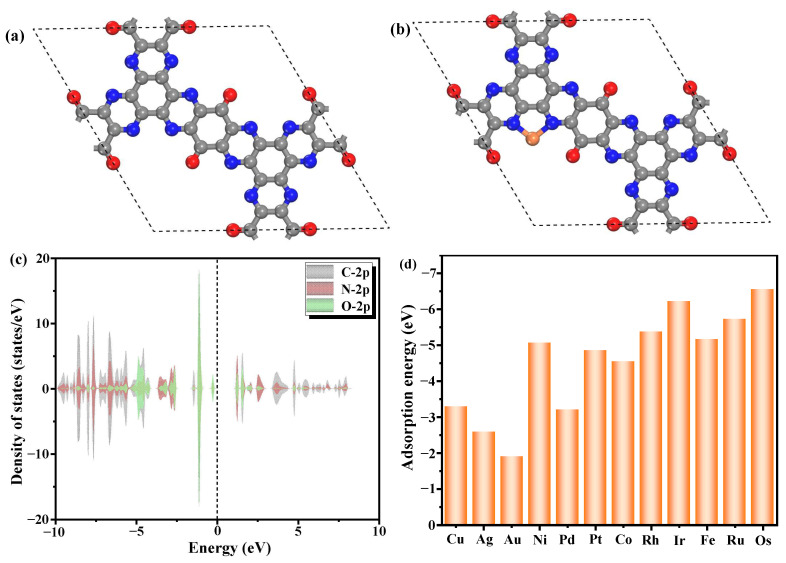
Optimal structures of (**a**) TQBQ-COF; (**b**) TM/TQBQ; (**c**) density of states of TQBQ-COF, and (**d**) adsorption energy of TM/TQBQ. The gray, blue, red, and light orange spheres represent C, N, O, and TM elements, respectively.

Figure 1a illustrates the stable geometric configurations of the pristine TQBQ-COF. The unit cell of TQBQ-COF consists of 48 atoms (30 C atoms, 12 N atoms, and 6 O atoms). The optimized lattice parameters of TQBQ-COF are *a* = *b* = 16.71 Å and *γ* = 120°, which aligns with the previous work (*a* = *b* = 16.70 Å) [32] and confirms the accuracy of our computational results. DOS analysis reveals that the valance band maximum and conduction band minimum of TQBQ-COF are mainly contributed by the 2p orbital of N and O. Charge density difference and Bader charge analysis (Figure S1) further reveal localized electron accumulation near N and O atoms, underscoring their role as preferential anchoring sites for TM atoms. Therefore, on this basis, we have constructed a model of the TM/TQBQ, as depicted in Figure 1b. In the present study, twelve kinds of TM atoms (Cu, Ag, Au, Ni, Pd, Pt, Co, Rh, Ir, Fe, Ru, and Os) were selected for the construction of SACs. These atoms have been commonly used to design SACs for the OER and ORR in previous works [33,34]. Prior to studying the catalytic activities of TM/TQBQ toward the OER and ORR, we first explored the adsorption energy (*E*_ad_) of different TM atoms, because the strong interaction between TM atoms and substrates is the basic premise of its long-term catalytic application. The calculated *E*_ad_s of TM/TQBQ are represented in Figure 1d. One can find from Figure 1d that the *E*_ad_s of all of the considered systems are relatively negative, indicating their excellent thermodynamic stability. Moreover, with the increase of the number of holes in the d shell, *E*_ads_ demonstrate a gradual upward trend, indicating that the interaction between metal atoms and substrates is enhanced. That is to say, the stability of SACs is closely related to the electronic configuration of the supported metal atoms, which lays the foundation for the design of stable and efficient SACs. To gain a deeper understanding of the interaction between TM atoms and substrates, we performed a Bader charge analysis, as shown in Figure S2. The remarkable electron transfer (0.52–1.28 e^−^) provides strong evidence for the stability of the catalyst. Of particular interest is the strong linear correlation between Δ*q* and *E*_ad_ (Figure S3), indicating that the amount of charge transfer between metal atoms and substrates is the primary determinant of adsorption strength. Additionally, it is worth mentioning that this correlation does not show a completely straight line. This relationship aligns with an “accept-donation” mechanism, where charge redistribution at the interface governs adsorption strength.

### 3.2. Catalytic Performance for OER and ORR

**Figure 2:**
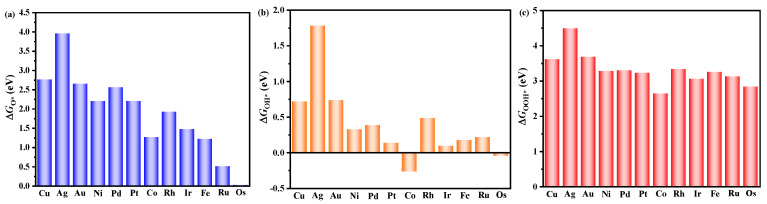
Adsorption free energies of (**a**) ∆GO*; (**b**) ∆GOH* and (**c**) ∆GOOH*.

**Figure 3:**
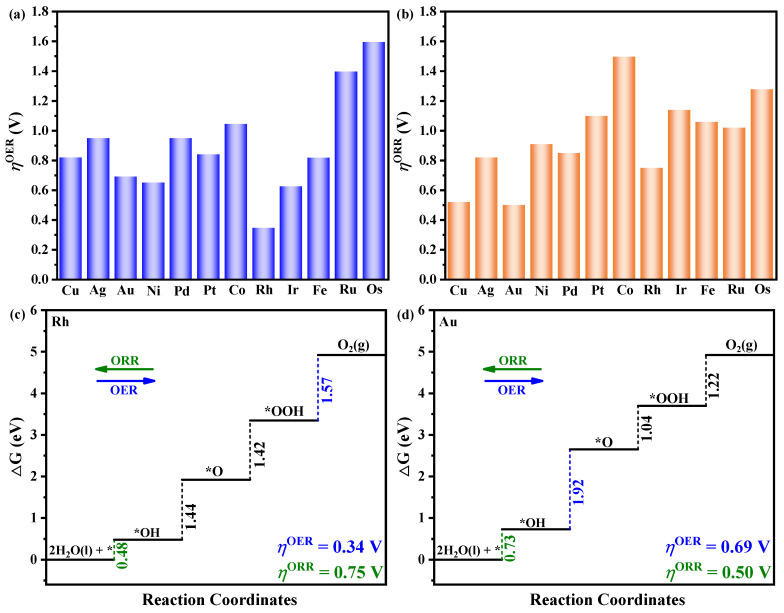
(**a**) ∆*G*_O*_ and (**b**) ∆*G*_OH*_ of TM/TQBQ; Free energy diagrams of OER and ORR processes for (**c**) Rh/TQBQ and (**d**) Au/TQBQ. (* represents the substrate).

The OER process was delineated as comprising four fundamental steps: (i) H_2_O (*l*) → OH* + H^+^ + e^−^; (ii) OH* → O* + H^+^ + e^−^; (iii) O* + H_2_O (*l*) → OOH* + H^+^ + e^−^; (iv) OOH* → O_2_ (*g*) + H^+^ + e^−^. The ORR process is regarded as the inverse reaction of the OER process. The assessment of OER and ORR activity hinges on the variation of Gibbs free energy (ΔG) at each reaction step under the equilibrium electrode potential. According to the Sabatier principle, both excessively strong and excessively weak adsorption of reaction intermediates onto catalysts impede the progression of OER and ORR, which emphasizes the importance of an optimal adsorption strength. To comprehensively evaluate the OER/ORR catalytic performance of TM/TQBQ, we primarily calculated the adsorption Gibbs free energy of each reaction intermediate (∆*G*_O*_, ∆*G*_OH*_, and ∆*G*_OOH*_), just as plotted in Figure 2. Notably, the variations of ∆*G*_O*_, ∆*G*_OH*_, and ∆*G*_OOH*_ across all systems are consistent. Relatively, cobalt group elements and iron group elements have smaller values of ∆*G*_O*_, ∆*G*_OH*_, and ∆*G*_OOH*_. This phenomenon is predominantly governed by the electronic configuration of the TM atoms. Specifically, the unsaturated d orbitals of cobalt and iron group elements facilitate easier electron transfer with reaction intermediates, leading to enhanced adsorption onto these intermediates. Conversely, the fully occupied d orbitals of copper group elements typically result in weaker adsorption of intermediates. A more negative value of ∆*G*_O*_, ∆*G*_OH*_, and ∆*G*_OOH*_ signifies a stronger interaction between the intermediate and the catalyst. However, too strong adsorption also hinders the conversion to the subsequent reaction intermediate. For an ideal OER and ORR process, the ∆*G* of each elementary reaction should be 1.23 eV. To better evaluate the OER/ORR catalytic activity of TM/TQBQ, we constructed a reaction free energy diagram based on the calculated ∆*G*_O*_, ∆*G*_OH*_ and ∆*G*_OOH*_ (Figure 3c,d and Figure S4). The potential determining step (PDS) of OER is shown in blue for OER and denoted in green for ORR. For copper and nickel group elements, the PDS of OER involves the transformation from OH* to O*. Conversely, for iron group elements, the PDS of OER is the conversion from O* to OOH*. This divergence in PDS is mainly due to the change of the adsorption energy of intermediates. As for ORR, the PDS involves the conversion of O_2_ to OOH* or OH* to H_2_O. In order to gain a deeper understanding of the activity disparities between different systems, the overpotentials of OER (*η*^OER^) and ORR (*η*^ORR^) for all systems are plotted in Figure 3a,b. Among all of the considered systems, Rh/TQBQ is predicted to be the best OER catalyst with an *η*^OER^ of 0.34 V, outperforming the benchmark IrO_2_ (110) catalyst (0.52 V) [35]. Moreover, Au/TQBQ demonstrates superior ORR catalytic performance with an *η*^ORR^ of 0.50 V, followed by Au/TQBQ (*η*^ORR^ = 0.52 V). The *η*^ORR^ values close to that of Pt (111) (*η*^ORR^ = 0.48 V) suggest their promising potential as ORR catalysts [36].

### 3.3. The Origin of Catalytic Activity

**Figure 4:**
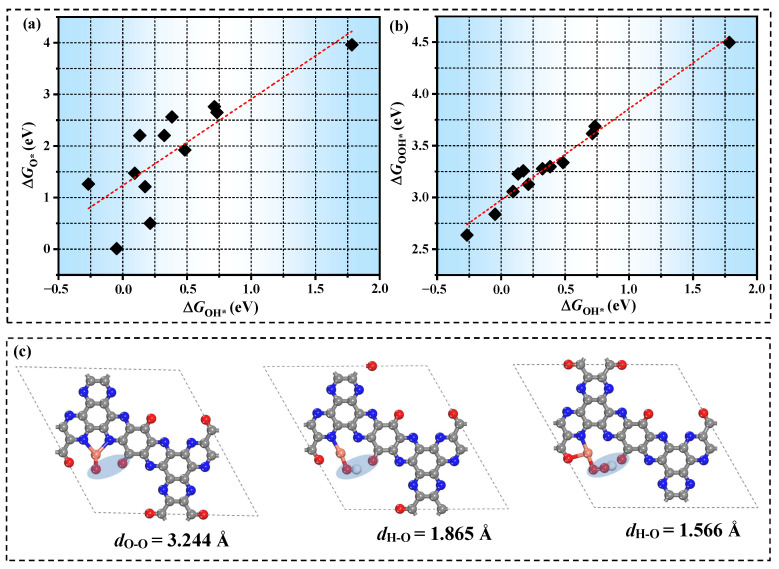
Scaling relationships between the (**a**) ∆*G*_OH*_ and ∆*G*_O*_; (**b**) ∆*G*_OH*_ and ∆*G*_OOH*_; (**c**) the optimal structures of O, OH, and OOH intermediate adsorption on Cu/TQBQ.

As mentioned above, the ∆*G* plays a important role in determining OER/ORR catalytic performance. Consequently, elucidating the relationship between adsorption free energy and catalyst activity is of utmost importance. To gain deeper insights into the origin of catalytic activity for TM/TQBQ, the scaling relationship between ∆*G*_O*_, ∆*G*_OH*_, and ∆*G*_OOH*_ was investigated, just as shown in Figure 4a,b. The unity of active sites for the intermediates on SACs results in a strong linear correlation between their adsorption energies, as observed in numerous prior studies [37,38]. This scaling relationship between the adsorption energies of intermediates becomes a major obstacle to the design of efficient OER and ORR catalysts. Therefore, overcoming the limitations imposed by this scaling relationship is crucial for advancing the design of novel and effective catalysts. In this work, as can be seen in Figure 4a,b, compared with the perfect linear proportional relationship between ∆*G*_OH*_ and ∆*G*_OOH*_, the correlation between ∆*G*_OH*_ and ∆*G*_O*_ remains worse. An important means to break through the limitation of scaling relationships is to change the adsorption behavior of different intermediates. To further comprehend the variations in adsorption energy correlations among different intermediates in this work (see Figure 4a,b), such as Cu/TQBQ for example, we plotted the adsorption configuration of O, OH, and OOH intermediates in Figure 4c. It is evident that the interaction between adjacent O atoms and the O intermediate is weak, with a large O-O bond length of 3.244 Å. However, due to the existence of H atoms in OH and OOH intermediates, there is a strong interaction between these two intermediates and the O atom on the TQBQ boundary, and the O-H bond lengths are 1.865 and 1.566 Å, respectively. The distinct impacts of boundary O atoms on the adsorption of O and OH intermediates serve as the underlying cause for their poor adsorption energy correlation. This discovery will provide a novel perspective for overcoming the scaling relationship limitations in OER and ORR processes. In addition, the DOS for various SACs has been thoroughly investigated, as demonstrated in Figure S5. For all systems, one can find that the Fermi level is mainly composed of TM d orbitals and N and O 2p orbitals. This observation underscores the robust interaction between TM and N/O atoms, which indicates the source of the catalyst stability. Additionally, compared to the pristine TQBQ-COF, the introduction of TM atoms results in the electronic states of TM/TQBQ becoming more continuous and crossing the Fermi level. This suggests that the TM/TQBQ exhibits metallic behaviors, which are beneficial for the electrochemical reaction process. Subsequently, we explored the scaling relationship between the d-band center (*ε*_d_) and ∆*G*_O*_/∆*G*_OH*_/∆*G*_OOH*_. On the whole, there is an obvious negative correlation between the *ε*_d_ and the ∆*G*_O*_/∆*G*_OH*_/∆*G*_OOH*_, as depicted in Figure S6. In other words, a closer proximity of *ε*_d_ to the Fermi level is associated with stronger metal-intermediate interactions.

**Figure 5:**
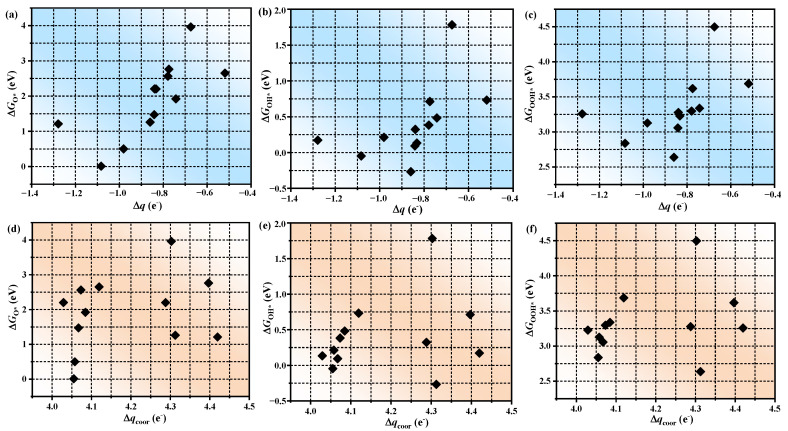
Scaling relationships between the ∆*q* and (**a**) ∆*G*_O*_; (**b**) ∆*G*_OH*_; (**c**) ∆*G*_OOH*_. Scaling relationship between the ∆*q*_coor_ and (**d**) ∆*G*_O*_; (**e**) ∆*G*_OH*_; (**f**) ∆*G*_OOH*_.

The adsorption strength of each intermediate on various TM atoms exhibits significant variability, indicating that ∆*G* has a strong correlation with the electronic configuration of the central metal atom. Therefore, based on Bader charge analysis, we explored the relationship between the charge of TM atoms (*q*) and ∆*G*, as depicted in Figure S7. While a nominal correlation exists between *q* and ∆*G*, the pronounced scatter renders q an inadequate descriptor for catalytic activity. Subsequently, we discussed the correlation between ∆*q* and ∆*G*, as shown in Figure 5a–c. Intriguingly, an inverted volcanic curve emerged between ∆*q* and ∆*G*. The charge transfer between the metal atom and the substrate decreases, which demonstrates that the charge supply ability of the central metal atom is weak, resulting in weaker adsorption of intermediates. Therefore, compared with the total charge of outer electrons of TM atoms, the charge transferred from TM atoms to the substrate can better reflect the differences in catalyst activity. To delineate the contribution of coordination atoms, scaling relationships between the charge transfer of coordinating atoms (∆*q*_coor_) and ∆*G* were established (depicted in Figure 5d–f). Notably, ∆*q*_coor_ exhibits superior correlation with ∆*G* compared to ∆*q*, unequivocally affirming the pivotal role of coordination environments in modulating catalytic behavior. Therefore, taking Au/TQBQ as an example, we deeply investigated the charge transfer of distinct moieties in the process of the adsorption of various intermediates (Figure S8). Overall, O acquires the largest electrons, followed by OH, and finally OOH. However, it is noteworthy that the trend of charge transfer in intermediates is not completely consistent with that of TM atoms. Such disparity underscores the active participation of the coordination matrix—particularly boundary oxygen atoms—in mediating electron transfer during intermediate adsorption, consistent with prior studies emphasizing the critical influence of oxygen vacancies on catalytic regulation [39,40]. These findings emphasize the great potential of an unsaturated coordination environment in SAC performance regulation.

### 3.4. The Prediction of Catalytic Performance

**Figure 6:**
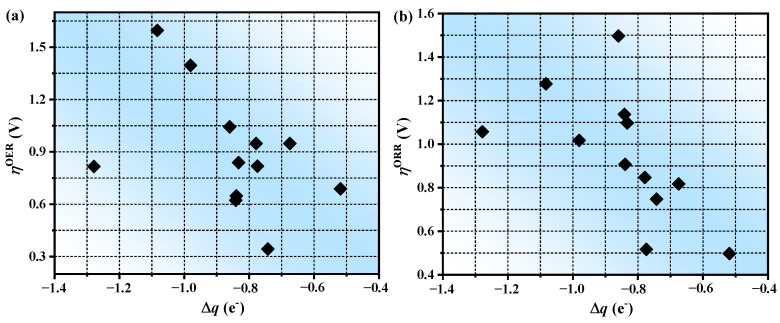
Scaling relationships between the ∆*q* and (**a**) *η*^OER^; (**b**) *η*^ORR^.

The conventional trial-and-error approach to catalyst design remains effective. However, it also exhibits significant costs. To address this limitation, a descriptor-based rational design strategy has been proposed. By selecting one or several properties of materials and connecting them with their catalytic performance, the complex calculation model can be simplified to a low-dimensional one, which is described by a series of reactivity descriptors. Our systematic investigation of ∆*q* revealed a distinct volcano-shaped relationship with *η*^OER^/*η*^OER^, as illustrated in Figure 6. More charge transfer will lead to the enhancement of the adsorption of intermediates, which makes the transformation of reaction intermediates difficult. However, excessive charge accumulation will result in the rejection of intermediates by active centers, which will lead to weak adsorption of intermediates and may improve the catalytic performance. Consequently, the existence of a volcanic relationship between ∆*q* and *η*^OER^/*η*^OER^ shows the practicability of ∆*q* as a reliable activity descriptor.

**Figure 7:**
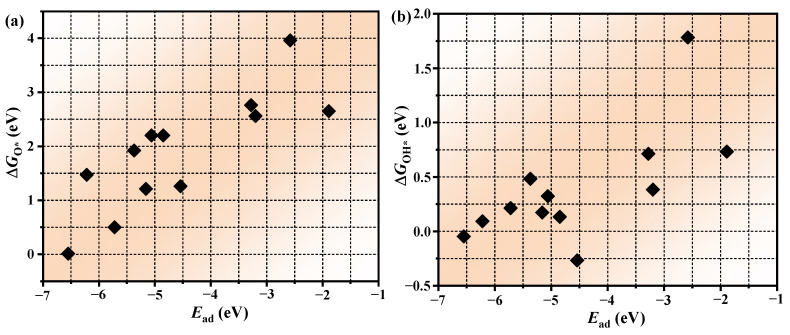

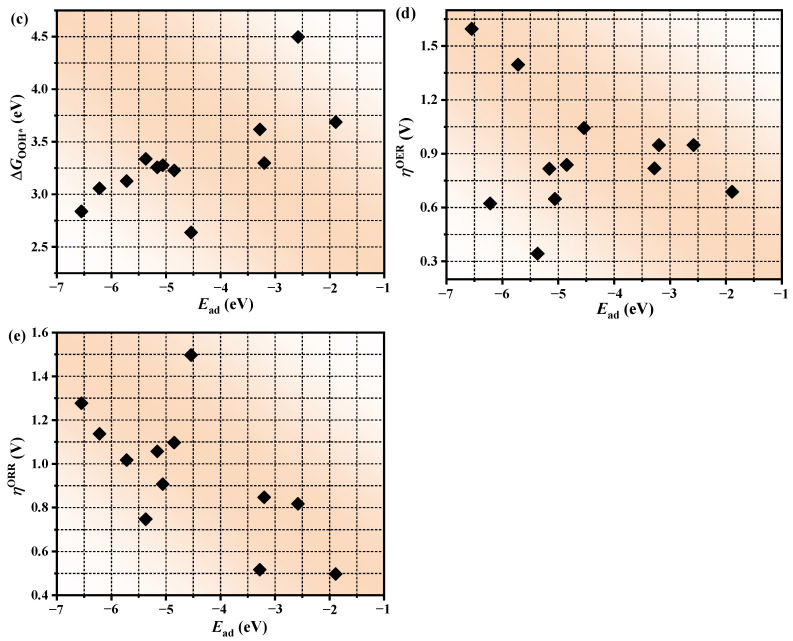
Scaling relationships between the *E*_ad_ and (**a**) ∆*G*_O*_; (**b**) ∆*G*_OH*_; (**c**) ∆*G*_OOH*_; (**d**) *η*^OER^; (**e**) *η*^ORR^.

Complementing our previous demonstration of *E*_ad_ as a predictive descriptor for transition metal dichalcogenide-supported single-atom catalysts (TMD-SACs) in OER [41], we further elucidated the fundamental connection between *E*_ad_ and ∆*G*, as depicted in Figure 7a–c. The results reveal that there is a positive correlation between *E*_ad_ and ∆*G*, indicating that the stronger the adsorption of TM atoms, the stronger the adsorption of reaction intermediates. Subsequently, we constructed the relationship between *E*_ad_ and *η*^OER^/*η*^ORR^, as shown in Figure 7d,e. Obviously, there is a clear negative correlation between *E*_ad_ and *η*^OER^/*η*^ORR^, enabling the development of a simplified activity prediction model. This breakthrough allows catalytic performance evaluation through single-parameter *E*_ad_ calculations, circumventing computationally intensive simulations of full reaction pathways. Based on this approach, the screening of efficient OER/ORR catalysts can be significantly accelerated.

## 4. Conclusions

In conclusion, the OER and ORR catalytic performance of TM/TQBQ was comprehensively studied using DFT calculations. The negative adsorption energies for these systems verify the stability of TM atoms anchored on TQBQ. We verified that the Gibbs free energy of intermediate adsorption on TM-TQBQ can be used as a robust descriptor for predicting OER/ORR activity. Notably, excellent correlations were observed between ∆*q*, *E*_ad_, and the *η*^OER^/*η*^ORR^, which can prove their reliability and effectiveness in predicting the OER/ORR catalytic performance of COF-based SACs. Among the candidates, Rh/TQBQ exhibits remarkable OER activity with a low overpotential of 0.34 V, surpassing commercial IrO_2_ benchmarks. Au/TQBQ demonstrates exceptional ORR activity with an overpotential of 0.50 V, indicating its potential for application in efficient electrocatalysis. The outstanding OER and ORR activities of Rh/TQBQ and Au/TQBQ position them as promising catalysts for rechargeable Zn-air batteries. Their low overpotentials directly translate to reduced energy loss during charge-discharge cycles, enhancing round-trip efficiency. More importantly, a critical breakthrough lies in the pivotal role of boundary oxygen atoms in TQBQ, which disrupt conventional scaling relationships for OER/ORR intermediates, offering new avenues to circumvent intrinsic thermodynamic limitations in electrocatalysis. Our results provide a more efficient and simpler method for the rational design of efficient SACs based on COFs.

## Data Availability

The original contributions presented in this study are included in the article/Supplementary Material. Further inquiries can be directed to the corresponding authors.

## Supplementary Materials

The following supporting information can be downloaded at: https://www.mdpi.com/article/10.3390/molecules30071505/s1, Figure S1: (a) Bader charge (e^−^) and (b) charge density difference of the TQBQ-COF. The yellow isosurface (9∗10^−3^ e/Å^3^) represents electron accumulation and the blue isosurface (9∗10^−3^ e/Å^3^) represents electron depletion. Figure S2: Bader charge of the TM-TQBQ. Figure S3: Scaling relationship between the Δ*q* and *E*_ad_. Figure S4: Free energy diagrams of OER and ORR processes based on TM/4N-GR. Figure S5: Density of states of TM-TQBQ. Figure S6: Scaling relationship between the d-band center and (a) ∆*G*_O_; (b) ∆*G*_OH*_ and ∆*G*_OOH*_. Figure S7: Scaling relationship between the *q* and (a) ∆*G*_O_; (b) ∆*G*_OH*_ and ∆*G*_OOH*_. Figure S8: (a) Schematic diagram of adsorption intermediates divided into different moieties and (b) Δ*q* of different moieties.
