# Supplementary material for: Rational Design of Covalent Organic Frameworks-Based Single Atom Catalysts for Oxygen Evolution Reaction and Oxygen Reduction Reaction
Source: Molecules. 2025 Mar 28;30(7):1505. doi: 10.3390/molecules30071505 (PMC11990586; doi:10.3390/molecules30071505)
Supplement: Supplementary file 1 [file molecules-30-01505-s001.zip › molecules-3460290-supplementary.pdf]

# Rational design of covalent organic frameworks-based single atom catalysts for OER and ORR

Wenli Xie[a], Bin Cui[b], Desheng Liu [b], Haicai Huang [c, d *], Chuanlu Yang [e *]

[a] *School of Materials Science and Engineering, Guangdong Ocean University, Yangjiang 529500, China.*

[b] *School of Physics, State Key Laboratory of Crystal Materials, Shandong University, Jinan 250100, China.*

[c] *Chongqing Institute of Green and Intelligent Technology, Chinese Academy of Sciences, Chongqing 400714, China*

[d] *Chongqing School, University of Chinese Academy of Sciences, Chongqing 400714, China.*

[e] *School of Physics and Optocelectronic Engineering, Ludong University, Yantai 264025, China.*

The adsorption energies of TM atoms ($E_{ad}$) are defined using the following formula:

$$E_{ad}=E_{total} - (E_{sub}+E_{TM})$$

where $E_{total}$ represents the total energy of the system, $E_{total}$ denotes the energy of the substrate, and $E_{TM}$ corresponds to the energy of the adsorbed TM atom. This definitionimplies that a more negative value of Ead indicates a more stable adsorption structure.

Additionally, the Gibbs free energy was calculated utilizing the appropriate formulas:

$$G=E_{DFT}+E_{ZPE}+\int C_p d_T - TS$$

*Corresponding author.

*E-mail address: huanghaicai@cigit.ac.cn; ycl@ldu.edu.cn.*

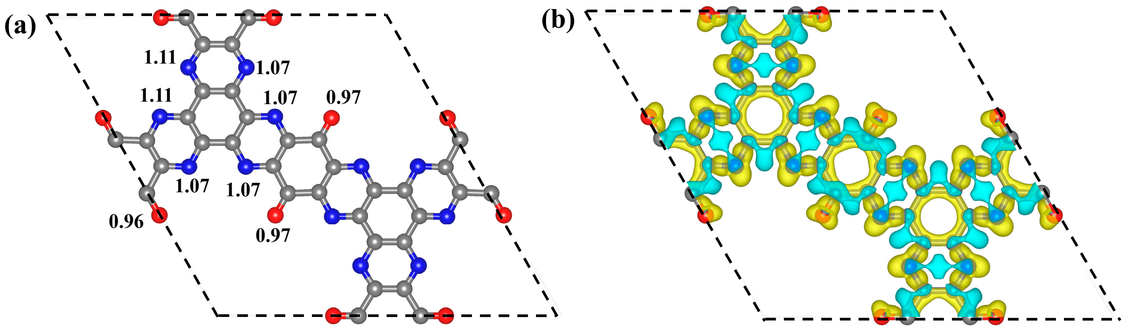


**Figure S1** (a) Bader charge (e$^-$) and (b) charge density difference of the TQBQ-COF. The yellow isosurface (9*10$^{-3}$ e/Å$^3$) represents electron accumulation and the blue isosurface (9*10$^{-3}$ e/Å$^3$) represents electron depletion.

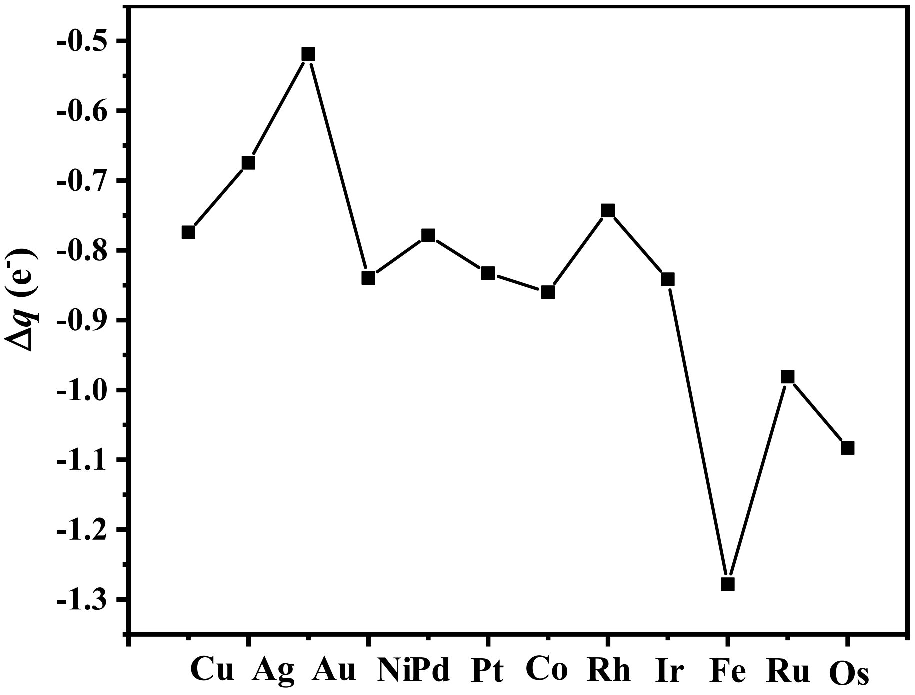


**Figure S2** Bader charge of the TM-TQBQ.

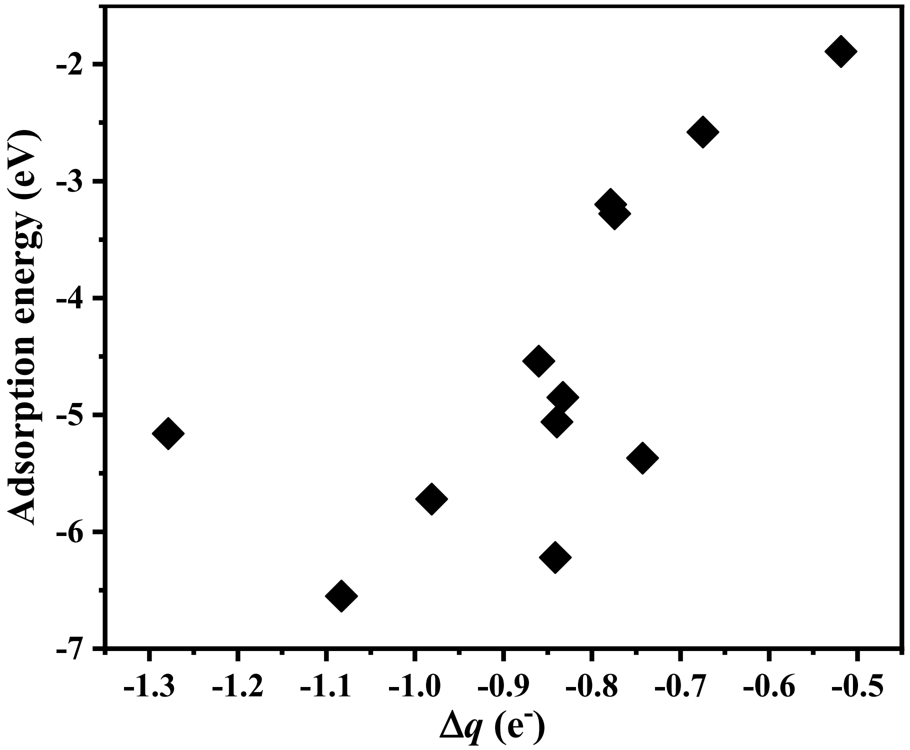


**Figure S3** Scaling relationship between the $\Delta q$ and $E_{\mathrm{ad}}$.

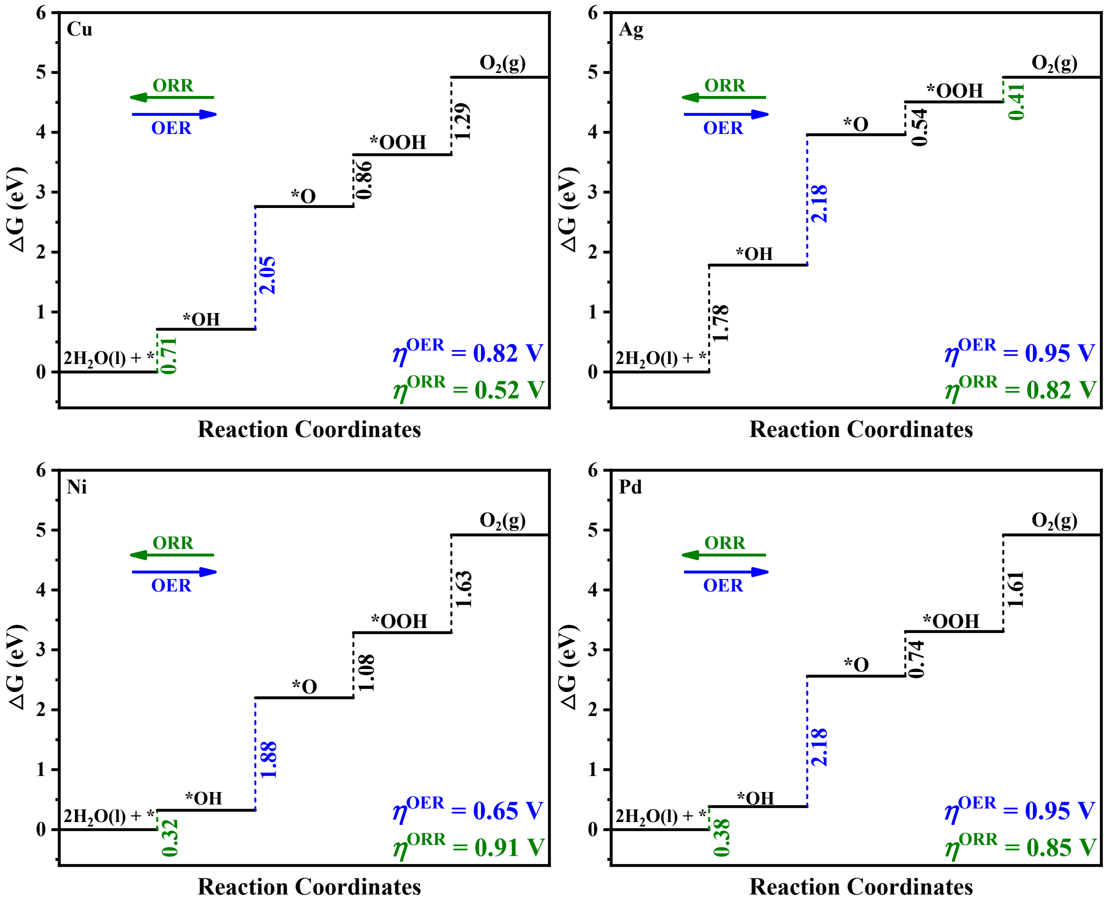

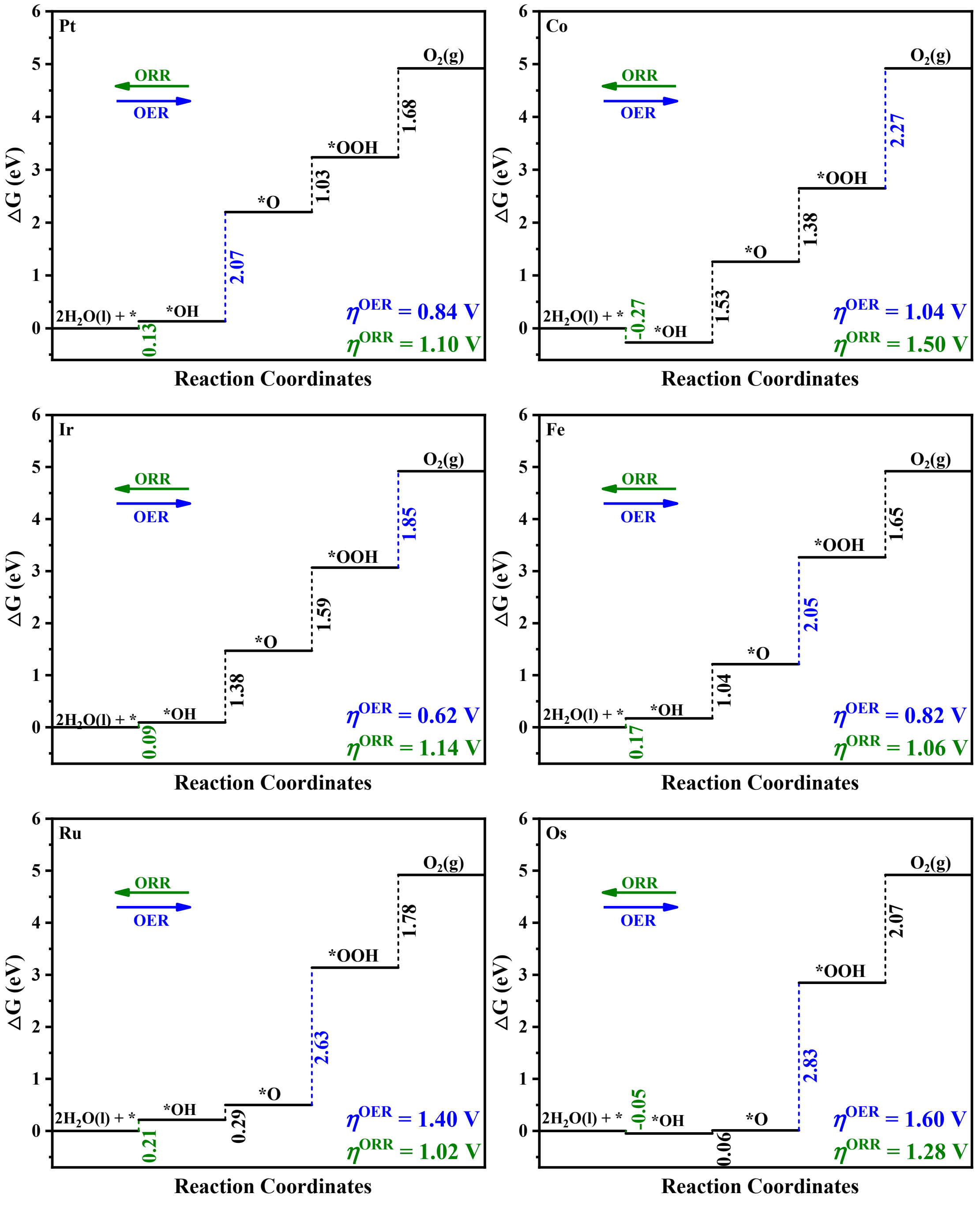


**Figure S4** Free energy diagrams of OER and ORR processes based on TM/4N-GR.

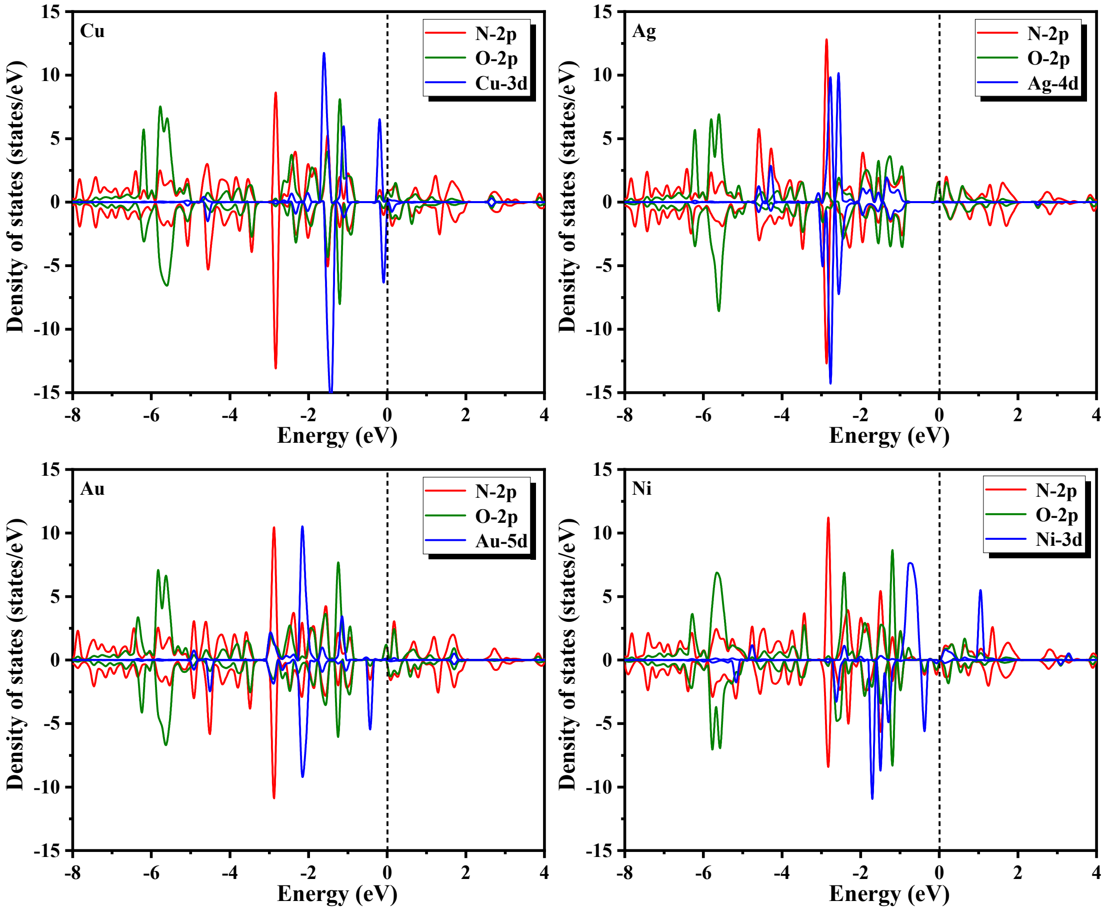

Cu
N-2p
O-2p
Cu-3d
Ag
N-2p
O-2p
Ag-4d
Au
N-2p
O-2p
Au-5d
Ni
N-2p
O-2p
Ni-3d
Density of states (states/eV)
Energy (eV)

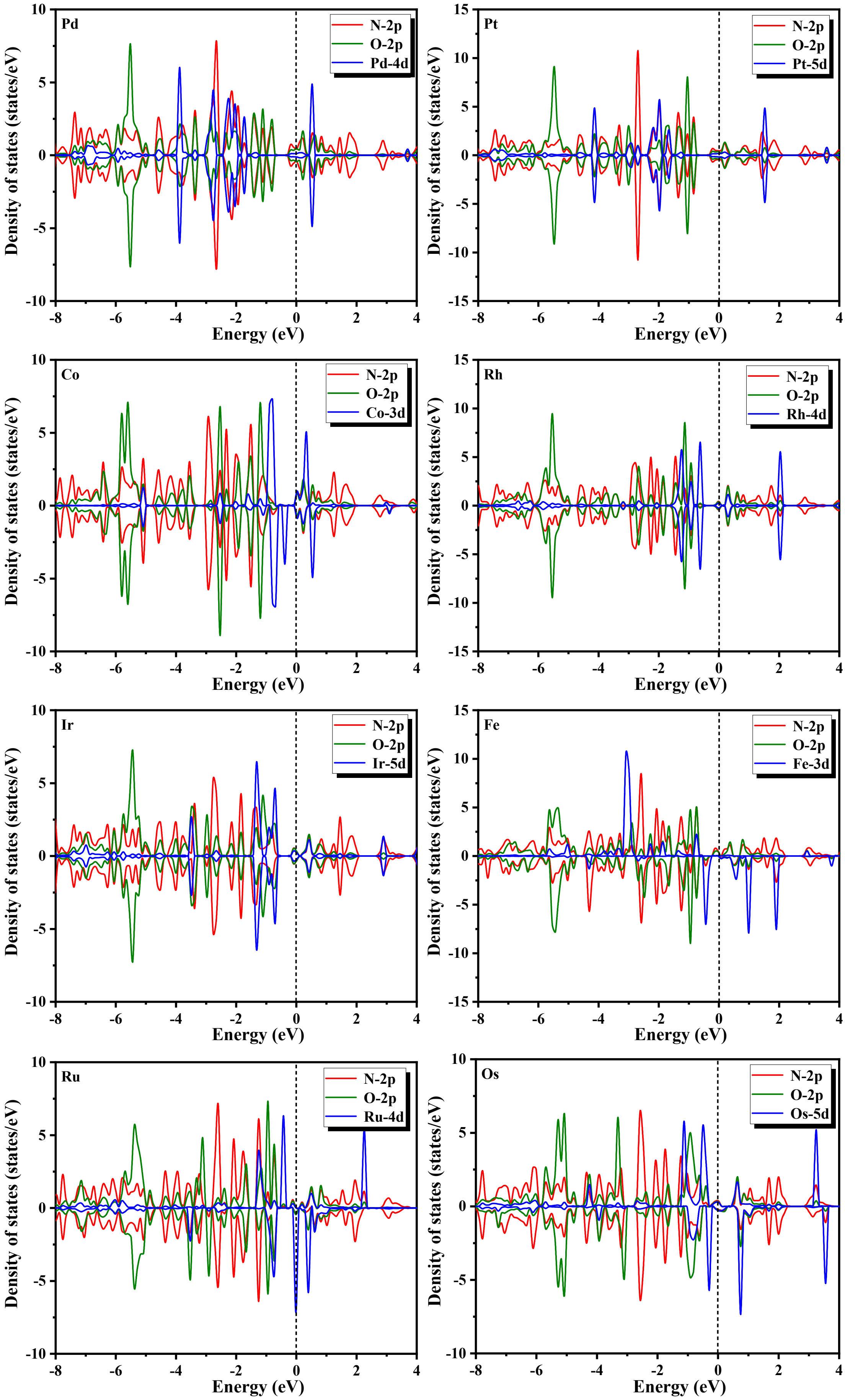

Pd
N-2p
O-2p
Pd-4d
Density of states (states/eV)
Energy (eV)
Pt
N-2p
O-2p
Pt-5d
Density of states (states/eV)
Energy (eV)
Co
N-2p
O-2p
Co-3d
Density of states (states/eV)
Energy (eV)
Rh
N-2p
O-2p
Rh-4d
Density of states (states/eV)
Energy (eV)
Ir
N-2p
O-2p
Ir-5d
Density of states (states/eV)
Energy (eV)
Fe
N-2p
O-2p
Fe-3d
Density of states (states/eV)
Energy (eV)
Ru
N-2p
O-2p
Ru-4d
Density of states (states/eV)
Energy (eV)
Os
N-2p
O-2p
Os-5d
Density of states (states/eV)
Energy (eV)

**Figure S5** Density of states of TM-TQBQ.

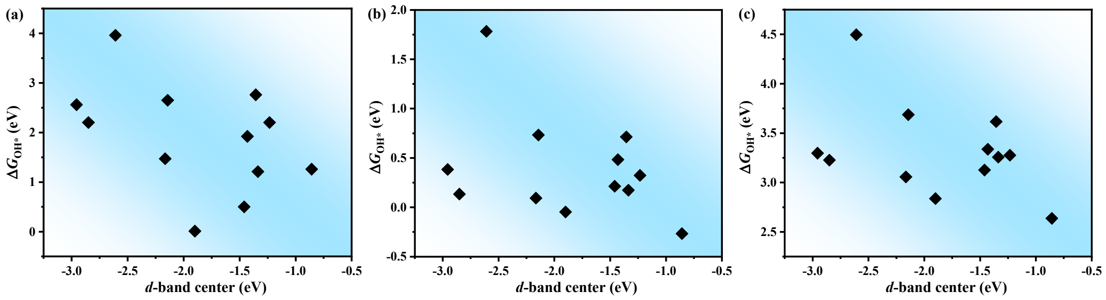


**Figure S6** Scaling relationship between the d-band center and (a) $\Delta G_{O}$; (b) $\Delta G_{OH*}$ and $\Delta G_{OOH*}$.

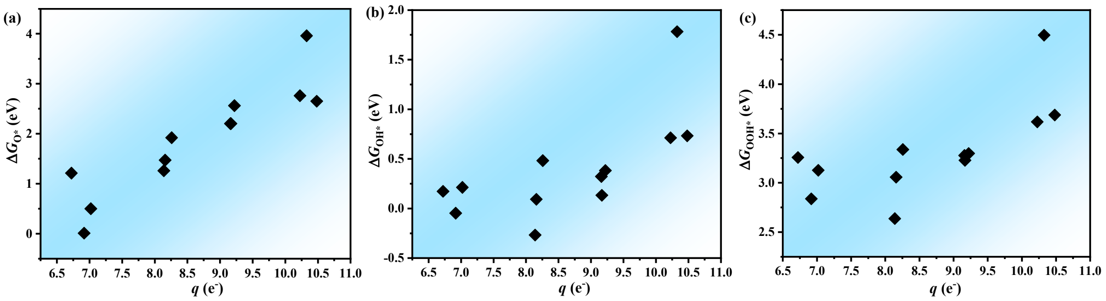

**Figure S7** Scaling relationship between the $q$ and (a) $\Delta G_{O}$; (b) $\Delta G_{OH*}$ and $\Delta G_{OOH*}$.

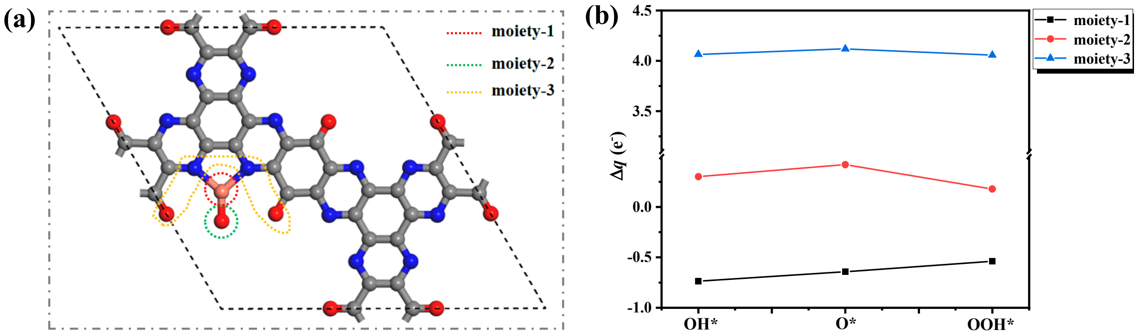


**Figure S8** (a) Schematic diagram of adsorption intermediates divided into different moieties and (b) $\Delta q$ of different moieties.
